# Exploring attitudes of adolescents and caregivers towards community-based delivery of the HPV vaccine: a qualitative study

**DOI:** 10.1186/s12889-020-09632-2

**Published:** 2020-10-09

**Authors:** Shoba Ramanadhan, Constance Fontanet, Marina Teixeira, Sitara Mahtani, Ingrid Katz

**Affiliations:** 1 Harvard T.H. Chan School of Public Health, 677 Longwood Avenue, Kresge 7th floor, Boston, MA 02115 USA; 2 Brigham and Women’s Hospital, 75 Francis Street, Boston, MA 02115 USA; 3 Dana-Farber Cancer Institute, 450 Brookline Ave, Boston, MA 02215 USA; 4 Harvard Global Health Institute, 42 Church St, Cambridge, MA 02138 USA

**Keywords:** Human papillomavirus, HPV, Cancer prevention, Racial and ethnic minorities, Community-engaged research, Adolescents, Vaccination, Community settings

## Abstract

**Background:**

Human Papillomavirus (HPV) vaccination among adolescents is an important strategy to prevent cervical and other cancers in adulthood. However, uptake remains far below the Healthy People 2020 targets for the US. Given the barriers to population-level vaccination policies and challenges to incorporating additional action items during clinical visits, we sought to explore alternative delivery mechanisms, specifically delivery of the vaccine in community settings.

**Methods:**

We conducted six focus groups (three with adolescents aged 11–14 who had not received the HPV vaccine and three with caregivers of adolescents meeting those criteria) from Black, Latino, and Brazilian communities in Massachusetts. We utilized a framework analysis approach that involved a multi-stage coding process employing both prefigured and emergent codes. Initial interpretations were refined through consultation with an advisory board.

**Results:**

Adolescents and caregivers expressed a range of concerns about the HPV vaccine and also described interest in learning more about the vaccine, emphasizing the importance of a relationship with a trusted provider as a facilitator of vaccine acceptance. Regarding community-based delivery of the vaccine, reactions were mainly negative. However, adolescents and caregivers noted that receiving information in community settings that could seed a conversation with a trusted provider would be welcome. Interestingly, the notion of a trusted provider seemed to extend broadly to practitioners linked to the trusted main provider.

**Conclusions:**

The study highlights an opportunity for increasing HPV vaccination among some racial and ethnic minority populations by leveraging trusted community organizations to provide information and seed conversations with a potentially broad group of trusted providers. A task-shifting approach, or reliance on staff with fewer formal credentials, may offer opportunities to support vaccination in resource-constrained settings.

## Background

Each year, the Human Papillomavirus (HPV) causes about 34,800 new cancer cases in the United States [1]. HPV vaccination among adolescents offers an important opportunity to prevent cervical and other cancers in adulthood [2]. However, across the US, HPV vaccine uptake among adolescents remains low, with only 54% of 13–17 years old completing the recommended course of the vaccine as of 2019. This uptake is far below the goal set by the federal government (Healthy People 2020) of 80% coverage among adolescents aged 13–15 years and rates of uptake of other routine child/adolescent vaccines [3, 4]. Policy solutions, such as the national HPV vaccination program for young people in Australia, have been highly effective, though the lack of political will around this solution suggests this may be a longer-term goal in the US [5, 6].

A range of provider-level interventions offers promise in increasing HPV vaccination rates, from prompting providers to recommend the vaccine to implementation of reminder-recall systems in the electronic health record [7–11]. This focus is fitting, given that the majority of children in the US receive vaccinations from pediatricians and family physicians [12]. However, implementation has been restricted by competing demands for the clinical visit, provider knowledge gaps, concerns related to reimbursement, and preferences regarding which adolescents to vaccinate (based on age and gender) [13]. Additionally, some providers have expressed discomfort with addressing HPV vaccination as the potentially sensitive and time-consuming resulting discussion may take time from other concerns they view as a higher priority [14]. These challenges are compounded by barriers among adolescents and caregivers, e.g., concerns about vaccine safety and efficacy and low perception of risk of HPV infection, though provider recommendation has been shown repeatedly to be a major motivator for vaccination [13, 15].

Given the challenges related to provider-focused interventions to increase HPV vaccination, it may be useful to consider other systems for vaccine delivery. The US Preventive Services Task Force highlights community-level action (in combination with other activities) as important drivers of vaccine uptake. Although the literature on HPV interventions is still limited, the Task Force highlights opportunities to increase vaccinations broadly by increasing access to services, increasing community demand, utilizing provider- or systems-level interventions, or combining strategies [16]. We focus here on opportunities to increase access, with an emphasis on reducing logistical barriers that prevent community members from getting the care they are seeking. Seeking new solutions, researchers have highlighted the potential to deliver the HPV vaccine through schools and pharmacies, which has been effective for influenza vaccines [17–20]. In a few qualitative studies, parents were willing to consider vaccinating their children outside of the medical home, and therefore these settings may represent additional opportunities for HPV vaccination [21, 22]. School-based vaccination has been effective in other countries. However, it is currently limited in the US due to reimbursement issues and limited availability of school-based health centers, two critical drivers of HPV vaccination rates [23].

We focus here on opportunities to raise coverage levels to target goals among adolescents from three communities of color to ensure that the innovation of interest – the HPV vaccine – reaches maximal impact among these young people. This is critical as adolescents who are racial and ethnic minorities are less likely than their White counterparts to complete the vaccine series [24] and additionally, Black and Latina adult women tend to be diagnosed with later stage cervical cancer and have higher mortality rates than non-Hispanic White women [25, 26] Given the importance of focusing on racial and ethnic minorities, we had three broad areas of inquiry. First, what is the context in which HPV vaccination decisions are made by adolescents and caregivers? Second, how do adolescents and caregivers react to a proposed strategy of delivering the HPV vaccine in community settings? Third, what other delivery strategies might adolescents and caregivers suggest?

## Methods

### Study context

Data come from formative research to design an intervention to increase HPV vaccination rates by engaging with community organizations that serve racial/ethnic minorities and low socioeconomic status populations. The effort was undertaken in consultation with the Outreach Core of the U54 Partnership between the Dana-Farber/Harvard Cancer Center and the University of Massachusetts, Boston. The Outreach Core uses a community-based participatory research approach, working in partnership with a Community Advisory Board [27]. The present study was conducted using a consultative model, in which the Outreach Core and Community Advisory Board members (full list in acknowledgments) were asked to share expertise at strategic points, but were not asked to engage in a fully participatory process [28].

The Outreach Core addresses the cancer-related needs of the Black community in Boston, the Latino community in Lawrence, and the Brazilian community in Greater Boston. Census data from 2019 highlights the diversity of the three groups served [29]. Boston has the largest population, with approximately 694,583 residents, 45% of whom identified as White, non-Hispanic; 25% as Black, non-Hispanic; 20% as Hispanic; and 10% as Asian, non-Hispanic. Lawrence had about 80,376 residents, 80% of whom identify as Hispanic; 14% as White, non-Hispanic; 5% as Black, non-Hispanic; and 2% as Asian, non-Hispanic. In Boston and Lawrence, respectively, 20 and 24% of the population live below the poverty line. The Brazilian community in Greater Boston is spread out across several cities, with recent data estimating the number of Brazilians to be about 30,600 in Middlesex County, which includes the Greater Boston area. Although individuals in the US who identify as having Brazilian ancestry tend to have higher levels of education and employment than other ethnic subgroups, important potential drivers of health inequities for the Boston-area Brazilian community include limited English proficiency and barriers to accessing healthcare related to immigration status [30, 31]. Importantly, each of the project’s partner communities has a vibrant community health sector, which includes a range of local organizations taking progressive, multi-sectoral action to address health inequities and social determinants of health. Limits in vaccination reporting prevent presentation of detailed estimates of HPV vaccination levels for these communities.

Young people in Massachusetts can receive state-supplied vaccines (including the HPV vaccine) up until their nineteenth birthdays regardless of insurance status [32, 33]. Additionally, uninsured children can get access to insurance through MassHealth, which is Massachusetts’ version of Medicaid [34]. In Massachusetts, minors need parental consent to receive the HPV vaccine, though legislation to allow minors to self-consent has been proposed. At this time, the main requirement is the delivery of the vaccine information sheet to the parent/caregiver [35].

### Conceptual framework

The study draws on a model of HPV vaccine acceptance developed by Katz and colleagues, which highlights the ways in which structural/sociocultural factors (e.g., access to vaccination and history of vaccination) and environmental factors (e.g., norms and governmental messages) influence adolescent-level factors (e.g., developmental characteristics and healthcare utilization) as well as caregiver-level factors (e.g., beliefs, and relationship with adolescent) to drive vaccination behavior. The model emphasizes that the critical influences shift between the decision to vaccinate and later completion of the series [36]. We also included an emphasis on the interactions between adolescents, caregivers, and providers to our framework, given the important role providers play in HPV vaccination. Finally, we drew on concepts of implementation science to support the exploration of community-based delivery of the HPV vaccine. We considered the proposal to shift the delivery of the vaccine to more highly accessible settings (community settings) with potentially less-burdened staff to be a test of a new implementation strategy, or method to support integration of an evidence-based innovation (here, the HPV vaccine) [37].

### Participants and data collection

Data were collected through focus group discussions, which are a useful method for using group interactions to elicit shared norms and perspectives from participants [38]. We focused on adolescents aged 11–14 who had not received the HPV vaccine yet and caregivers of adolescents meeting those criteria and collected data in the fall of 2018. Experienced, doctoral-level qualitative researchers facilitated the discussions. The discussions were held in accessible community locations, e.g., the offices of a community organization in a neighborhood near participants’ homes, and were audio-recorded. A notetaker was present and took supplementary notes. Participants were recruited through partner organizations and health-focused coalitions and with the support of our advisory board, mainly using email and word of mouth. Separate groups were run for caregivers and adolescents, and the groups were additionally segmented according to cultural group and geography: Black residents of Boston, Latinos in Greater Lawrence, and Brazilians living in Greater Boston. Understanding opportunities to increase HPV vaccination in these groups can support broader goals to ensure equity in reaching Healthy People 2020 targets. We selected this age group intending to target adolescents who would need to complete two doses of the vaccine [39]. The clinical guidance at the start of the study suggested 11 years as the starting point for vaccination, though we recognize the vaccine is now also recommended starting at age 9 [40].

Three focus group discussions were held with individuals who identified as Black, Latino, and/or Brazilian and were caregivers to children aged 11–14 who had not yet received HPV vaccination to their knowledge. All discussions were moderated by researchers with doctoral-level training in sociology and fluency in the language(s) used for discussion. The groups included four, six, and nine participants. The discussion among Black caregivers in Boston was conducted in English, the one among Latino caregivers in Lawrence in English and Spanish, and the one among Brazilian caregivers in Boston in English and Brazilian Portuguese. The discussions took approximately 90 min. The focus group discussions followed a semi-structured interview guide that drew on the conceptual framework, the literature around HPV vaccination and evidence-based strategies for scaling cancer prevention interventions, and consultations with the advisory board. The major topic headings and a summary of the vignette are presented in Table 1; the guide for caregivers is available as a supplemental file.

**Table 1:** Topic headings for the interview guides and a summary of the vignette

**Topic Area**	
Processes by which healthcare decisions are made in the family	
Competing demands that can interfere with healthcare	
Sentiment towards vaccination in general	
Perception of HPV vaccination	
Reasons for not initiating vaccination, including any relevant interactions with providers	
Perception of healthcare delivery in alternative settings	
Feedback on community-based delivery strategies as a response to the vignette	
**Vignette summary**	
The vignette centered around Ana and Diego, 11-year-old twins from the town in which the discussion was conducted. They have heard about the HPV vaccine in the past, but their mother was concerned it could be harmful and one of the twins was worried about the needle. Today, while with their mother at a health fair in the park, they saw a brief video about the HPV vaccine and liked what they learned, especially that the vaccine can prevent cancer. Now they can get the first shot at the health fair.	

The second set of focus group discussions were conducted with adolescents who identified as Black, Latino, and/or Brazilian, were aged 11–14 years, and had not been vaccinated for HPV (based on caregiver report). Discussions were conducted in English and were moderated by doctoral-level qualitative researchers (including IK, SR, and one of the researchers mentioned above). The discussions took approximately 70 min and included five, eight, and nine participants. The focus group discussions followed a similar semi-structured interview guide to the one described above but emphasized the adolescent perspective; the guide is included as a supplemental file. Some of the adolescents and caregivers were related to each other, e.g., mother-child pairs. This decision greatly supported the recruitment of participants from families with many competing demands for their time. Drawing on concepts of information power [41], we estimated a small number of focus groups would meet our goals of exploring initial reactions to a novel implementation strategy among adolescents and caregivers from the three partner communities, leveraging a conceptual framework, and conducting a pragmatic analysis.

### Data analysis

A professional transcription service transcribed (and translated as needed) interview and focus group audio recordings. Transcripts were analyzed and summarized by a team of four trained researchers. The interdisciplinary team included an implementation scientist (SR) and clinician-scientist (IK), with doctoral degrees in public health and medicine, respectively, as well as two student researchers (MT and CF), who were trained in qualitative methods. Three team members have been trained in implementation science and recognized that this training tends to emphasize the importance of increasing the adoption of evidence-based practices. However, the team has experience with community-engaged research related to health promotion in communities experiencing cancer inequities and is sensitive to the range of influences that determine acceptance of guidelines. Additionally, a consultation process with the community advisory board offered an opportunity to check and correct for team biases.

The analysis was guided by a framework analysis approach and involved a multi-stage coding process that utilized both prefigured and emergent codes [42]. The four-member team jointly developed the coding scheme, starting with prefigured codes based on domains of interest in the moderator guides (Table 1), followed by a more inductive and open-coding process to identify emergent codes in the data [43]. Transcripts were initially coded by two student researchers (MT and CF), with coding review and mediation of disagreements by a senior researcher (SR). This team of three then conducted additional coding to support the analysis and development of additional codes. Relevant to this paper, the emergent codes mainly related to the reactions to the community-based delivery strategy, discussion of trusted providers / care settings, and responses that related to cultural and social factors unique to the participants’ communities. We initially analyzed data separately for the adolescents and caregivers and then integrated the analytic process to support comparisons between adolescents and caregivers. These methods were enhanced by the use of NVivo 11 [44]. Early interpretations of the data were shared with the Community Advisory Board and refined based on their feedback.

### Ethics approval

All study procedures and documents were approved by the Institutional Review Board of the Harvard T.H. Chan School of Public Health. The team collected consent forms from the adults and paired consent/assent forms from adolescents and their parents/caregivers.

## Results

A total of 19 individuals participated in the caregiver focus group discussions; all were parents of at least one child aged 11–14 who had not received the HPV vaccine (to their knowledge). All identified as female; 7 identified as Latino, 8 as Black, and 4 as Brazilian. A total of 22 individuals aged 11–14 participated in the adolescent focus group discussions. Of this group, 14 identified as female and 8 as male; 12 as Latino, 2 as Black, 2 as White, 1 as Asian/Pacific Islander, and 5 as Brazilian. As summarized in Table 2, important insights were offered regarding adolescent and caregiver perceptions of vaccines (general and HPV-specific), the information environment, family decision-making processes, and responses to a proposed community-based delivery strategy.

**Table 2:** Domains/key themes related to context for HPV vaccination, reactions to community-based delivery, additional suggestions

Domains	Key themes
1.Vaccination decision-making context	
1.A. Vaccine-related concerns	Adolescents had mainly negative reactions regarding vaccines (generally and specifically for the HPV vaccine), with concerns about pain and anxiety related to anticipating the shot, as well as side effects. Caregivers had mixed reactions, with concerns about side effects (and potential long-term impact on the reproductive health of young women receiving the vaccine) and positive expectations about the protective benefits of the vaccines. They also voiced an additional concern related to the reproductive health of young women who have received the HPV vaccine.
1.B. Information environment	Adolescents noted wanting information about HPV, including reassurance around vaccination (particularly from a trusted source). Their primary sources of information included parents, doctors, and the internet. Caregivers expressed interest in additional information, emphasizing a need for a balance between the pros and cons of receiving the HPV vaccine. Trusted sources included doctors, the internet, family, and friends.
1.C. Family decision-making processes	Both adolescents and caregivers identified parents as the primary drivers of general healthcare decisions and vaccination specifically, with variation in the extent to which adolescents were involved. For HPV, there was more discussion of consulting outside trusted sources.
1.D. Importance of a trusting relationship with the provider	Both adolescents and caregivers emphasized the need to know and trust the provider, though adolescents noted that the vaccine was acceptable as long as their parents trusted the provider.
2. Community-based vaccine delivery strategy	
2.A. Concerns raised	The negative reactions centered on the receiving the HPV vaccine in community settings and mainly related to concerns around engaging with an unknown provider.
2.B. Opportunities highlighted	The positive reactions were mainly related to opportunities for outreach and engagement. Caregivers were interested in attending educational events focused on HPV, particularly those geared towards adolescent health. They also wanted additional information on both the positive and negative consequences of the vaccine.

### Vaccine-related concerns

Adolescents had mixed reactions to the idea of vaccines, broadly, and emphasized concerns more than perceived benefits. Their main concerns related to the pain of the injection and the negative experience of anticipating the shot, as well as possible side effects, including temporary sickness. Of the positive reactions about vaccines, they mainly focused on prevention and opportunities to avoid disease.

*“When I have to get vaccines, I think of flu shots. And flu shots have pros and cons. They get – sometimes they get you even more sick, which that’s why I don’t – I prefer not to get the flu shot. But people tell me that it works, guys. I know I get sick for a little bit, but then I heal …it’s hard to believe.” (Adolescent FGD3).*

Specific to HPV, the potential to prevent cancer was highlighted as an important selling point for the vaccine.

For vaccines overall, caregivers voiced a great deal of concern about the potential for vaccines to either cause the illness they are meant to prevent or have serious side effects. They also noted that in many cases, they did not have a choice about vaccination, e.g., in the context of school- or sports-related requirements. Participants also discussed the idea that vaccines are useful for preventing disease, albeit with less emphasis than the concerns raised related to vaccines. Specific to HPV, there was also attention to long-term side effects, particularly related to the reproductive health of young people who have received the HPV vaccine.

*“I listen to friends talking about it…Oh, you’re doing that for your daughter? No, because later they don’t get [to] have a family.” (Caregiver FGD1).*

### Information environment related to vaccines

There was some debate among adolescents regarding what information would be helpful to promote vaccination. Some adolescents expressed that their fears and concerns would not easily be attenuated, while some reported that knowing about the positive effects of vaccines and being reassured about the side effects (especially if it was by a trusted source) would help them decide to get the vaccine. Adolescents had four main trusted sources for information on vaccines (presented from most to least commonly emphasized): doctors, the internet, parents, and other trusted adults. Adolescents described respect for the knowledge their doctors had and appreciation for the ways in which providers explained the benefits of vaccination to them. Regarding internet-based sources, adolescents reported seeking information on vaccines online and many referenced using search functions, such as Google or Siri. Others referred to social media, including YouTube and Facebook. Adolescents commonly referenced parents as an important source of trusted information, including in the context of serving as intermediaries between the doctor and the adolescent. In one focus group, participants noted that their parents were unable to easily understand what was being said during medical appointments because of language barriers. Finally, adolescents referred to other trusted adults as sources, ranging from health teachers to family members (particularly those with health training).

Caregivers overwhelmingly reported that they lacked information about the goals and the side effects of the vaccines offered to their children. There was a strong consensus that additional information was always helpful, particularly if it explained the benefits of the vaccine as well as possible negative consequences. They highlighted two of the same sources of information as adolescents: the adolescent’s doctor and web-based sources. They emphasized the importance of going to a trusted provider, such as the adolescent’s doctor. A few respondents expressed strong distrust of health care providers, which often stemmed from previous negative experience. When discussing online sources, many participants mentioned using Google to search for information, but one group had an extensive discussion about the variable quality of the information found through these searches and potential anxiety and confusion produced by such searches. One caregiver also brought up the influence of their cultural community:

*“…[W]e end up influencing other people in the community where we are, which is the same thing* *you* *are doing… When we are together in a place, I feel safe, don’t you think? They won’t lie to me.” (Caregiver FGD3).*

### Family decision-making

For both general health and HPV-focused decisions, adolescents and caregivers both identified parents as drivers of the decisions, often in consultation with the adolescent or with an openness to questions, but typically with the caregiver driving the process.

*“As long as my parents approve it…I just go with my parents. I trust them, so it’s better...Sometimes, I don’t like to make decisions. It’s hard.” (Adolescent FGD3).*

For caregivers, when considering decisions on HPV, there was more need for outside, reliable sources. The main outside sources that influenced caregivers on vaccinating against HPV were family members and trusted providers.

*“The first thing I’ll do is go to my mother. Then the doctor.” (Caregiver FGD3).*

### The importance of a trusted provider

Adolescents highlighted trust in their provider as an important driver of vaccination; interestingly that trust may be direct from the adolescent to the provider, or indirect as the parents trust the provider. Overwhelmingly, adolescents stated their preference that the vaccine be administered by a doctor or nurse they already knew.

For caregivers, trust and a lengthy relationship were important elements of the vaccine acceptance process. Importantly, the sense of trust accrued to specific providers or care teams (e.g., doctors, nurses, and other providers at the site linked to their main provider) was a direct contrast to a lack of trust in the broader medical system and health information environment. Caregivers mostly felt a trained professional who is known to them should administer vaccines to their children, and some specifically noted that they would not be comfortable with an unknown person, e.g., at a pharmacy delivering vaccines.

*“It’s hard because – yeah, it’s the pharmacy and I just don’t trust anyone with my kids. I don’t know those people. Every time I go to my pharmacy, there’s somebody different there. And I just – I don’t know what they’re giving my kid. They could be giving my kid something else.” (Caregiver FGD2).*

Caregivers in the Brazilian group also noted the influence of norms in Brazil as having impact on healthcare decision-making. Some adults in this group mentioned that their children received much of their initial healthcare in Brazil and continue to be seen by providers in Brazil when they visit.

### Community-based vaccine delivery strategy

Overall, participants had a negative reaction to the scenario proposed in the vignette, in which the first shot in the HPV vaccine series was offered in a community setting. There was limited feedback in the focus group discussions regarding alternative delivery strategies; instead, the conversations focused more on opportunities to use community settings as a place for learning more about the vaccine.

### Concerns raised related to community-based delivery

Overall, negative reactions to the community-based strategy related to receiving outside traditional medical settings. Adolescents described a sense that their peers would not be sufficiently interested in health-related issues to engage with such an offering in a community setting. They also raised concerns about pain and fear of vaccines and also noted a distrust of vaccine delivery outside of the doctor’s office/by a professional other than their trusted provider.

*“If you were to give vaccines in … a community place… like not at a clinic, I think that’s, sorry to say, but kind of dumb.” (Adolescent FGD3).*

Among caregivers, most were firmly opposed to vaccine receipt outside of the doctor’s office, but some noted that they could be convinced under the right conditions such as cleanliness and accreditation. Caregivers also noted concerns about the likely disconnect between available materials and their needs. Specifically, they highlighted concerns about the materials in that they might a) be available in languages they could not read, b) would not be tailored for caregivers versus young people, and c) would not present complete information about potential side effects.

### Opportunities highlighted related to community-based delivery

Positive reactions to the community-based strategy were primarily driven by caregivers and related to opportunities for outreach and engagement. Specifically, caregivers focused on the benefits of attending educational events focused on HPV, particularly those geared towards adolescent health. Respondents noted that received information at a health fair allowed for more discussion and more time to review the information presented.

*“In [town] where I live there are a lot of churches that are strong in relation to immigrants there. In these places, people can go, talk and call, because they are in a place of safety. For example, in the church service, when people are there, or in a mass, afterwards you can talk and say there is a program like this. It works like this. There are professionals who talk…so this dissemination is important and a way to raise subjects and to explain in the language of the person in an environment where they feel secure.” (Caregiver FGD3).*

In addition, caregivers highlighted that receiving educational materials in the community provides more opportunities to promote conversations with a trusted provider in a clinic.

*“I am listening to what you’ve got to say. You know what I mean? But if I have – I might ask you a question or two, but I’m going to go back and ask my primary care to see if his answer is similar or conjunction to yours, just because whatever you tell me then and there is not going to say okay. I’m on. I’m going to do it.” (Caregiver FGD2).*

While adolescents expressed a reticence to engage with health programming in a community-based setting, they provided suggestions of ways to incorporate educational material into programming to make it more appealing to young people, by integrating with games, sports, opportunities to meet peers, connecting with schools, or having doctors share information. They also suggested incorporating narratives from young people to add authenticity to the information provided.

*“So if there was going to be a commercial, me personally, I would like kids to do it because almost every commercial is grownup and doctors talking about it. So I would want to hear it from doctors, but also kids, too.” (Adolescent FGD1).*

## Discussion

Broadly, the study points to an opportunity to better serve adolescents from three racial and ethnic minority communities by addressing misinformation about the HPV vaccine via local outreach to seed conversations with a trusted medical team. The trusted relationship appeared to be an important counterpoint to the fear adolescents expressed around receiving vaccines (both in terms of pain and uncertainty about the vaccine) and the concerns raised by caregivers. Community-based delivery of education, but not vaccination, was deemed acceptable and desired. The centrality of trusted providers and the importance of the information environment and local context were consistent with our underlying conceptual framework.

This inquiry assessed the acceptability of expanding access to HPV vaccination through community-based delivery of the vaccine, but our findings suggest that caregivers and adolescents were uncomfortable with the proposed scenario. They did not have much to say about alternative settings for delivery outside the clinic and instead emphasized community-based education related to HPV as a useful way to spark conversation about the vaccine with their trusted provider. Educational programs could address the misperceptions around vaccine side effects, such as the concern that vaccination would negatively impact young women’s reproductive health. The US Preventive Services Task Force is clear that community education alone is not a recommended strategy for increasing vaccination rates, but instead highlights the power of community education (and other demand-increasing strategies) in concert with expanded access to vaccination (such as home visits or extensions of the healthcare facility) [45, 46]. In this way, community education could provide the impetus and information needed to start a conversation with a provider.

As part of the educational outreach, our findings suggest that adolescents may respond better to direct acknowledgments of their concerns around pain and fear of the vaccination process, followed by placing these fears in the context of their broader goals. A previous review of the literature around communicating about the HPV vaccine pointed to the importance of letting patients and caregivers know about minor side effects associated with the vaccine, such as pain at the injection site. Avoidance of acknowledging these side effects can engender even greater distrust in the vaccine [47]. Caregiver trust in the medical provider was expected to increase the likelihood of vaccination because it could diminish adolescent fears and address caregiver concerns around safety. Although interest among caregivers and adolescents in receiving education is promising, our recent work and the work of others highlights the need to ensure that community-based practitioners view HPV as a priority topic and are comfortable conducting outreach and education regarding the vaccine, despite perceptions that it is a sensitive topic [18, 48].

The process of seeding community members with information to prime them for a discussion with providers may address the sticking point found in previous research by our team suggesting that clinicians were hesitant to start a conversation around HPV, as it could be a sensitive topic and could take valuable time away from other health issues. Having adolescents and their caregivers start the conversation (after having been exposed to it from a cancer prevention frame) may be an important opportunity. The customization of materials to meet the language needs of diverse communities is critical to this effort, as language barriers were raised repeatedly as important challenges to caregivers being able to get the information they sought or to take advantage of what their children were given by schools and other organizations. By better matching content and seeding conversations, caregivers and adolescents can benefit from shared decision-making, an approach in which clinical providers and patients engage with the best available evidence to collaboratively make decisions about care [49]. Some evidence has shown that involving and informing adolescents in shared decision-making regarding the HPV vaccine can affect uptake [50]. While facilitating these discussions has been shown to be hindered by parent and adolescent discomfort in regards to speaking about sex [51], the mechanism of shared decision-making may still prove useful and effective, particularly if families are given tools or engage with providers to promote these discussions.

Physician recommendation (using a presumptive frame and a clear recommendation for action) is a well-documented motivating factor for vaccination [9, 10, 52], and our findings extend this work, highlighting the power of provider recommendation because of the trust between the provider and the family, especially in a primary care setting where a long-lasting relationship can be formed. However, as we know from the literature, adding tasks to pediatricians’ and family physicians’ lists may not translate into increased vaccination [53]. An important opportunity may exist with the perception of a trusted medical home that emerged from the study. Both adolescents and caregivers reported willingness to have the shot delivered by another member of the clinical team, as long as the clinical site was one they knew and trusted. Thus, if the key is that the vaccination needs to happen within the clinical walls, there may be an opportunity to broaden the scope of the providers who deliver the shot. This idea of task-shifting, or having staff who are less formally credentialed take on the work of more formally credentialed staff, has been used to address a range of resource constraints in other public health and medical settings [54, 55].

As with any study, the results must be taken as a function of the strengths and limitations. Given the small scale of the project, we were able to gather high-level reactions to the proposed implementation strategy of community-based HPV vaccine delivery. Additionally, study participants were adolescents who had not been vaccinated or caregivers of adolescents who had not been vaccinated, but who were open to participating in a conversation about the vaccine. Therefore, the perspectives and processes uncovered may only apply to a particular subset of adolescents and caregivers. On the other hand, the study has three important strengths that outweigh the limitations. First, we focused explicitly on a diverse set of racial and ethnic minority groups that experience cancer inequities, with the intention of generating evidence that explicitly addresses the context in which underserved adolescents could receive HPV vaccination, a key opportunity to increase equity [56]. Second, consultation with our community advisory board strengthened our ability to incorporate context into the interpretations and decreased the risk of biased analysis. Third, the study leveraged a diversity of perspectives and included adolescent voices that are often left out of such inquiries [50].

## Conclusions

The study highlights an opportunity for increasing HPV vaccination in underserved communities that leverages the trust of community organizations to provide information and seed conversations with trusted providers. Future research can also attend to the potential we uncovered for a broad definition of “trusted providers” that leverages the ability to shift tasks to less credentialed providers who are part of a trusted clinical home. Exploration of such opportunities will be critical to ensure that racial and ethnic minority adolescents reach the Healthy People 2020 goal of 80% coverage. Learnings from such successes will be primed for adaptation and translation to other concerns for health promotion among racial and ethnic minority adolescents.

## Data Availability

The datasets generated and/or analyzed during the current study are not publicly available to protect the identity of respondents, but are available from the corresponding author on reasonable request.

## Abbreviations

HPV: Human Papillomavirus
FGD: Focus group discussion
